# Evaluation of the impact of six different DNA extraction methods for the representation of the microbial community associated with human chronic wound infections using a gel-based DNA profiling method

**DOI:** 10.1186/s13568-017-0477-z

**Published:** 2017-09-19

**Authors:** Ayomi Dilhari, Asanga Sampath, Chinthika Gunasekara, Neluka Fernando, Deepaka Weerasekara, Chris Sissons, Andrew McBain, Manjula Weerasekera

**Affiliations:** 1 Department of Microbiology, Faculty of Medical Sciences, University of Sri Jayewardenepura, Gangodawila, Nugegoda, Sri Lanka; 2 Department of Surgery, Faculty of Medical Sciences, University of Sri Jayewardenepura, Gangodawila, Nugegoda, Sri Lanka; 3 Department of Pathology and Molecular Medicine, University Otago, Wellington, New Zealand; 4 Faculty of Biology, Medicine and Health, The University of Manchester, Manchester, M13 9PT UK

**Keywords:** DNA extraction methods, Polymerase chain reaction-denaturing gradient gel electrophoresis (PCR-DGGE), Wound infection

## Abstract

Infected chronic wounds are polymicrobial in nature which include a diverse group of aerobic and anaerobic microorganisms. Majority of these communal microorganisms are difficult to grow in vitro. DNA fingerprinting methods such as polymerase chain reaction-denaturation gradient gel electrophoresis (PCR-DGGE) facilitate the microbial profiling of complex ecosystems including infected chronic wounds. Six different DNA extraction methods were compared for profiling of the microbial community associated with chronic wound infections using PCR-DGGE. Tissue debris obtained from chronic wound ulcers of ten patients were used for DNA extraction. Total nucleic acid was extracted from each specimen using six DNA extraction methods. The yield, purity and quality of DNA was measured and used for PCR amplification targeting V2–V3 region of eubacterial 16S rRNA gene. QIAGEN DNeasy Blood and Tissue Kit (K method) produced good quality genomic DNA compared to the other five DNA extraction methods and gave a broad diversity of bacterial communities in chronic wounds. Among the five conventional methods, bead beater/phenol–chloroform based DNA extraction method with STES buffer (BP1 method) gave a yield of DNA with a high purity and resulted in a higher DGGE band diversity. Although DNA extraction using heat and NaOH had the lowest purity, DGGE revealed a higher bacterial diversity. The findings suggest that the quality and the yield of genomic DNA are influenced by the DNA extraction protocol, thus a method should be carefully selected in profiling a complex microbial community.

## Introduction

Microorganisms associated with polymicrobial infections such as chronic wound infections are diverse and often unculturable (Han et al. 2011; Oates et al. 2012; Wadinamby 2013). Molecular biological methods based on 16S rDNA and other genetic markers have been used effectively to analyze diversity of microbial populations in environmental samples (Nakatsu 2007; Øvreås et al. 1997) as well as clinical specimens (Ariefdjohan et al. 2010; Oates et al. 2012; Walter et al. 2001). Molecular methods can bypass the culture isolation and can generate a comprehensive and precise set of data which is important to understand the role of these microorganisms in polymicrobial infections (Oates et al. 2012; Tannock 2002). As a molecular fingerprinting technique, polymerase chain reaction-denaturing gradient gel electrophoresis (PCR-DGGE) has been successfully applied to profile diverse microbial communities of various clinical specimens (Ariefdjohan et al. 2010; Oates et al. 2012; Walter et al. 2001; Weerasekera et al. 2017, 2013).

Extracting DNA from complex microbial communities is a challenge. The analytical success of molecular techniques is greatly affected by its reliance on the efficient cell lysis and the quality of the recovered DNA (Ariefdjohan et al. 2010; De Lipthay et al. 2004; McOrist et al. 2002). (De Lipthay et al. 2004; Moore et al. 2008). Successful extraction of DNA involves effective disruption of cells, denaturation of proteins and nucleoprotein complexes, inactivation of nucleases such as DNases and recovery of the DNA. The extracted DNA should have low contamination of proteins, carbohydrates, lipids and RNAs. The choice of the extraction method should be based on the required molecular weight of the target DNA, required quantity, purity, extraction time and cost (Ariefdjohan et al. 2010). Quality and integrity of the isolated nucleic acids will directly affect the results of the experiment. The goal of this study was to compare the relative efficacy of five conventional DNA extraction methods and one commercial kit in extracting bacterial genomic DNA from infected tissues of chronic wound specimens. Although these extraction methods have been separately reported for DNA extraction from several biological specimens (Ariefdjohan et al. 2010; Oates et al. 2012; Walter et al. 2001), our study further extends this knowledge by a direct comparison of these methods and application of the extracted DNA to PCR-DGGE technique. Further we aimed to identify the optimized DNA extraction conditions to obtain a high and pure DNA yield from chronic wound specimens in order to effectively profile bacterial communities using PCR-DGGE.

## Materials and methods

### Ethical statement

The study was granted ethical approval from Ethics Review Committee of University of Sri Jayewardenepura (Ref. No: 12/16) and Colombo South Teaching Hospital (Ref. No: 506) in Sri Lanka.

### Specimen collection and preparation

Wound tissue debridement specimens from chronic foot wounds were collected from ten patients undergoing surgical debridement at a Tertiary Care Hospital in Sri Lanka. The specimens were collected by a well-trained and qualified medical officer/surgeon after obtaining the informed consent from the patients.

The tissue debridement specimens were collected into sterile eppendorf tubes and immediately transported in a portable cooler and stored at −20 °C for a few weeks or −80 °C up to several months. The samples from each patient were kept separate and were not pooled. Each tissue debridement specimen was weighed and cut into 12 small pieces (each weighing 25 mg) using a sterile scalpel or a sharp sterile needle.

Twenty-five milligrams of tissue debridement specimen was subjected to each DNA extraction method. Each extraction was done in duplicate. The methods H1 and H2 involved heat treatment of tissue specimens (Asadzaheh et al. 2010; Sampath et al. 2016; Silva et al. 2012), BP1 and BP2 were based on bead beater–phenol chloroform extraction (Sampath et al. 2016; Walter et al. 2001; Weerasekera et al. 2013); method S was based on DNA precipitation at high salt concentration (Asadzaheh et al. 2010) and method K used a commercial DNA extraction kit (Oates et al. 2012).

### Heating in distilled water inside a boiling water bath (H1 method)

In the procedure of heat treatment in Distilled water (H1 method), the weighed tissue debridement sample was thoroughly minced and suspended in 100 µl of sterile distilled water. The specimen was immersed for 10 min in a 100 °C water bath. Tubes were centrifuged at (13,000 rpm) 15,493×*g* for 10 min and the supernatant was removed to a sterile tube and stored at −20 °C. This method was carried out as described by Silva et al. in 2012 with modifications (Silva et al. 2012).

### Heating in NaOH inside a boiling water bath (H2 method)

DNA extraction using the heat treatment in NaOH (H2 method) was carried out as described by Asadzaheh et al. (2010) with the modifications (Asadzaheh et al. 2010). Tissue debridement specimen was minced, suspended in 100 µl of 50 mM NaOH and incubated in a 100 °C water bath for 20 min. Subsequently 20 µl Tri-HCl (pH = 7.5) was added. The tube was gently mixed by inverting several times and centrifuged at (13,000 rpm) 15,493×*g* for 10 min. The upper aqueous phase was transferred into a sterile clean tube and stored at −20 °C until used.

### Bead beater–phenol chloroform extraction method using STES buffer (BP1 Method)

Bead beater–phenol chloroform extraction method using STES buffer (BP1 method) was carried out according to the procedure described by Sampath et al. in 2016 (Sampath et al. 2016). A tissue debridement specimen was suspended in 100 µl STES buffer [200 mM Tris HCl (pH 7.6), 100 mM EDTA, 0.1% SDS] and 40 µl of TE buffer [10 mM Tris HCl (pH 8), 1 mM EDTA]. Further, 120 µl Phenol: Chloroform mixture (1:1 V/V) and 0.3 g sterile zirconium beads (0.1 mm diameter; Bio Spec-Products) were added to each tube. Then the specimens were homogenized using a mini bead beater (model 3110BX; Bio Spec Products) at 480 rpm for 5 min. The upper aqueous phase (100 µl) was transferred to a sterile eppendorf tube. Ten microliter of 3 M sodium acetate was added and DNA was precipitated in the presence of 220 µl cold ethanol (100%) at −20 °C for 4 h. The solution was then subjected for centrifugation at (13,000 rpm) 15,493×*g* for 12 min. Air dried DNA pellet was dissolved in 30 µl TE buffer and stored at −20 °C until used.

### Bead beater–phenol chloroform extraction method using TN150 buffer (BP2 method)

The process of bead beater–phenol chloroform extraction method using TN150 buffer (BP2 method) was carried out according to the procedure described by Walter et al. (2001) with modifications (Walter et al. 2001). The weighed tissue debridement specimen was suspended in 1 ml of sterile TN150 buffer and 0.3 g of sterile zirconium beads (diameter, 0.1 mm) were added. The tube was placed in a mini-bead beater (model 3110BX; Bio Spec Products), shaken at 480 rpm for 3 min, and stored on ice. It was subjected for centrifugation at (13,000 rpm) 15,493×*g* for 5 min. The upper phase (300 µl) was transferred into a new sterile eppendorf tube. Two hundred microliters of saturated phenol and 200 µl of chloroform-isomyl alcohol (24:1) were then added. The tube was inverted several times and centrifuged at (13,000 rpm) 15,493×*g* for 12 min. The supernatant was transferred into a sterile new micro-centrifuge tube. Fifty microliters of 3 M sodium acetate and 1 ml ice cold ethanol was added to the tube, mixed by inverting and stored at −20 °C for 4 h for precipitation of DNA. Following centrifugation at (13,000 rpm) 15,493×*g* for 12 min, supernatant was discarded and the DNA pellet was allowed to air dry. After all the traces of alcohol had evaporated, DNA was dissolved in 30 µl of sterile TE buffer [10 mM Tris HCl (pH 8), 1 mM EDTA] and stored at −20 °C until used.

### Salting out method (S method)

Salting out method (S method) was performed according to the procedure described by Asadzaheh et al. (2010) with several modifications (Asadzaheh et al. 2010). The weighed tissue debridement sample was suspended in 600 µl of sterile TNES buffer [10 mM Tris–HCl pH = 7.5, 400 mM NaCl, 100 mM EDTA, 0.5% SDS] and 20 µl of proteinase K and mixed by inverting. The mixture was thoroughly mixed by vortex mixing and incubated at 50 °C inside a water bath until the tissue was completely lysed. After tissue debris was completely lysed, 200 µl 5 M NaCl was added and mixed vigorously for 20 s. The tube was centrifuged at (13,000 rpm) 15,493×*g* for 10 min and the supernatant was removed into a new sterile eppendorf tube. Equal volume of cold 100% ethanol was added, mixed, and kept overnight at −20 °C. The supernatant was discarded, after centrifugation at (13,000 rpm) 15,493×*g* for 12 min. The pellet was washed sequentially using 500 µl of 100% ethanol, 70% ethanol and allowed to air dry. The DNA was re-suspended with 30 µl TE buffer and stored at −20 °C until used.

### DNeasy blood and tissue kit (K method)

The DNA extraction using the DNeasy blood and tissue kit [Qiagen Ltd., West Sussex, United Kingdom] (K method) was carried out following the manufactures’ instructions. The weighed tissue debridement sample was dissected into small pieces and placed in a 1.5 ml micro-centrifuge tube. The dissected pieces of tissues were suspended in 180 µl of buffer ALT and 20 µl proteinase K. The mixture was thoroughly mixed by vortex mixing and incubated at 56 °C until the tissue was completely lysed. Following vortex mixing for 5 min, buffer AL (200 µl) was added to the sample and mixed. Two hundred microliter of ethanol was added. Mixture was pipetted into the DNeasy Mini Spin column placed inside a 2 ml collection tube and centrifuged at (8000 rpm) 5875×*g* for 1 min. The DNeasy Mini Spin column was then placed in a new collection tube and 500 µl of buffer, AW1 was added and centrifuged. This step was repeated with the buffer AW2 and a high spin was given to dry the column membrane. The DNeasy Mini Spin column was subsequently placed in a clean micro-centrifuge tube and 100 µl of buffer AE was added directly onto the membrane. Following centrifugation, DNA was eluted and stored at −20 °C until used.

### DNA quantification

DNA yield and DNA purity were determined using Nano drop 2000/200C spectrophotometer (Thermo Fisher Scientific, USA). The absorbance ratios; A260/280 nm and A260/230 nm were measured to assess DNA purity: A260/280 nm for protein contamination and A260/230 nm for salt and phenol contamination. DNA is known to absorb light at 260 nm and the A260/280 ratio; 1.8–2.0 and A260/230 ratio; >1.8 indicating that the sample was of good purity with little or no contamination (Vesty et al. 2017).

### PCR amplification of bacterial DNA

The V2–V3 region of the bacterial 16S ribosomal DNA (rDNA) was PCR amplified using previously published universal eubacterium-specific primers HDA1 (with additional GC clamp) (5′CGC CCG GGG CGC GCC CCG GGC GGG GCG GGG GCA CGG GGG GAC TCC TAC GGG AGG CAG CAG T 3′) (Forward primer) and HDA2 (5′ GTA TTA CCG CGG CTG CTG GCA C 3′) (Reverse primer) (Anukam and Reid 2007; Oates et al. 2012; Walter et al. 2001). This primer amplifies a DNA fragment having the nucleotide position between 339 and 539 (*E. coli* 16S rRNA gene). The GC-clamp, which is a sequence that is rich in guanine and cytosine, is added to the 5′ end of the forward primer in order to prevent DNA from being completely denatured into single strands and to improve band resolution in denaturing gels.

Amplification reactions were performed in 200 µl thin wall PCR^®^ tubes (BIOLOGIX, USA). For direct PCR reactions using HDA 1 (GC clamped) and HDA 2 primers, 50.0 µl reaction mixture consisted of 5.0 µl of 10× PCR buffer containing 25 mmol/l MgCl_2_ (Promega, USA); 1.0 µl of 10 mM dNTPs containing dATP, dGTP, dCTP and dTTP (Promega, USA); 1.0 µl of each primer [10 mM] (IDT, USA); 0.25 µl of Go Taq DNA polymerase (Promega, USA).

PCR amplification was done using GeneAmp PCR systems 9700 (Applied Bio systems). PCR reaction consisted of initial denaturation at 94 °C for 1 min, followed by 30 cycles consisting of 94 °C for 30 s for denaturation, 56 °C for 30 s for annealing and 72 °C for 30 s for extension, a final extension at 68 °C for 7 min with final hold at 4 °C. All PCR experiments included a negative (no template) control and a positive control. Resulting PCR products were separated by electrophoresis using 1× TAE [40 mM Tris HCl (pH 8), 20 mM acetic acid, 1 mM EDTA] on a 1.5% (w/v) agarose gel, stained with ethidium bromide and viewed by UV trans-illuminator [Vilber Lourmat, QUANTUM ST4].

### Denaturant gradient gel electrophoresis

Denaturant gradient gel electrophoresis of amplified PCR products which were generated from DNA extracted from wound tissue debridement samples were performed on acrylamide gels in a DCode™ universal mutation detection system (Bio-Rad) according to the conditions described by Rasiah et al. (2005). The gels were prepared using 8% acrylamide (acrylamide to bis-acrylamide, 37.5:1) with a 30–55% gradient of urea and formamide. The gels were run using 1x TAE buffer [40 mM Tris HCl (pH 8), 20 mM acetic acid, 1 mM EDTA] at a constant voltage of 130 V at 60 °C for 4 h. Electrophoresis buffer (1× TAE) was maintained throughout at 60 °C. Gels were stained with ethidium bromide, visualized and photographed on a UV trans-illuminator (Vilber Lourmat, QUANTUM ST4).

### Statistical analysis

All extractions were performed in duplicate to account for analytical variability. Means of DNA yield were analyzed using SPSS (version 20.0; Inc. Chicago) by one-way ANOVA with Welch correction. Data were expressed as mean ± SD. The extraction methods which have a significant difference between its mean values were grouped and multiple comparison was done using the Games–Howell. Differences were considered as significant when p value was <0.05.

## Results

All ten patients included in the study had chronic foot wound infections. The mean age of this group of patients were 64.4 years and the range was between 54 and 80 years. Male:female ratio was 1:1.

### Quality of DNA extracted each DNA extractions method

The differences between the protocols of the six DNA extraction techniques are described in Table 1. The yield, purity and quality of the genomic DNA obtained using the six DNA extraction methods from ten specimens are given in Table 2. The lower A260/280 ratio seen in DNA extracted from some methods may indicate the presence of protein, phenol, salts or other contaminants.

**Table 1:** Comparison of six different DNA extraction methods examined in this study

Extraction steps	BP_1_	BP_2_	K	H_1_	H_2_	S
Lysis buffer/agent	STES buffer	TN150 buffer	Tissue lysis buffer and protinase K	Distilled water	Aqueous NaOH	TNES buffer and protinase K
Cell lysis and homogenization	Bead beating	Bead beating	Incubation at 56 °C and vortexing	Boiling	Boiling	Incubation at 56 °C and vortexing
Extraction and DNA precipitation	Phenol chloroform and cold absolute ethanol	Phenol chloroform and cold absolute ethanol	Mini column and washing buffer	Heat	Heat	Hypertonic NaCl and cold absolute ethanol
Store in	TE buffer	TE buffer	Elution buffer	Distilled water	Aqueous NaOH	TE buffer
Approximate time for completion	7 ½ h	8 h	3 h	25 min	35 min	11 h

*BP*_*1*_ Bead beater phenol chloroform with STES buffer, *BP*_*2*_ Bead beater phenol chloroform with TN150 buffer, *K* DNeasy blood and tissue kit, *H*_*1*_ Heat treating in distilled water, *H*_*2*_ Heat treating in NaOH, *S* Salting out method

**Table 2:** DNA quality and average DNA yield obtained using different DNA extraction methods

	Patient/specimen No.									
	01	02	03	04	05	06	07	08	09	10
1 STES buffer and phenol chloroform extraction method (BP1)										
DNA yield (ng/µl)	48.65 ± 25.24	1747.95 ± 404.25	480.2 ± 359.07	195.25 ± 29.06	93.20 ± 31.25	937.75 ± 255.90	900.30 ± 639.79	1096.9 ± 451.7	226 ± 83.43	69.50 ± 10.89
A_260/280_	1.98 ± 0.08	1.80 ± 0.01	1.82 ± 0.06	1.88 ± 0.00	1.99 ± 0.02	1.88 ± 0.01	1.80 ± 0.01	1.82 ± 0.01	1.83 ± 0.02	1.96 ± 0.02
A_260/230_	0.87 ± 0.05	2.19 ± 0.07	1.75 ± 0.35	1.27 ± 0.02	1.05 ± 0.27	2.15 ± 0.01	1.82 ± 0.40	2.1 ± 0.11	1.69 ± 0.04	0.77 ± 0.05
2 TN150 buffer and phenol chloroform extraction method (BP2)										
DNA yield (ng/µl)	10.9 ± 1.56	310.05 ± 160.16	340.6 ± 12.45	88.35 ± 27.51	46.20 ± 21.64	274.05 ± 43.20	661.45 ± 500.70	402.25 ± 1.91	90.25 ± 76.44	93.70 ± 42.99
A_260/280_	2.07 ± 0.04	1.8 ± 0.03	1.77 ± 0.00	1.77 ± 0.04	1.73 ± 0.15	1.71 ± 0.02	1.86 ± 0.99	1.82 ± 0.01	1.8 ± 0.06	1.84 ± 0.12
A_260/230_	0.52 ± 0.01	1.84 ± 0.16	1.49 ± 0.10	1.15 ± 0.30	0.69 ± 0.17	1.57 ± 0.08	1.66 ± 0.32	1.79 ± 0.08	1.3 ± 0.44	1.37 ± 0.36
3 DNeasy blood and tissue kit (K)										
DNA yield (ng/µl)	39.45 ± 21.01	400.55 ± 447.25	698.2 ± 126.99	51.8 ± 28.56	50.75 ± 66.39	152.65 ± 13.08	718.1 ± 608.54	80.95 ± 0.49	316.25 ± 127.77	388.40 ± 120.49
A_260/280_	1.87 ± 0.05	1.80 ± 0.01	1.81 ± 0.01	1.78 ± 0.11	1.87 ± 0.17	1.80 ± 0.01	1.82 ± 0.01	1.8 ± 0.01	1.81 ± 0	1.84 ± 0.01
A_260/230_	2.1 ± 0.40	1.74 ± 0.66	2.17 ± 0.11	2.19 ± 0.06	1.56 ± 0.00	1.65 ± 0.37	2.15 ± 0.07	1.81 ± 0.11	1.82 ± 0.55	2.15 ± 0.06
4 Heating in distilled water (H1)										
DNA yield (ng/µl)	213.25 ± 46.46	51.65 ± 19.02	260.95 ± 70.36	72.2 ± 36.63	142.45 ± 47.73	277.6 ± 9.76	429.05 ± 4.60	145.6 ± 168.57	97.25 ± 73.89	255.35 ± 109.95
A_260/280_	1.22 ± 0.04	1.38 ± 0.06	1.26 ± 0.01	1.56 ± 0.05	1.36 ± 0.17	1.39 ± 0.05	1.41 ± 0.05	1.39 ± 0.05	1.41 ± 0.09	1.74 ± 0.18
A_260/230_	0.3 ± 0.04	0.28 ± 0.02	0.43 ± 0.00	0.56 ± 0.07	0.29 ± 0.02	0.32 ± 0.01	0.56 ± 1.01	0.33 ± 0.11	0.25 ± 0.04	0.71 ± 0.36
5 Heating in aqueous NaOH (H2)										
DNA yield (ng/µl)	131 ± 25.74	356.9 ± 189.22	606.95 ± 82.94	185.5 ± 32.81	146.8 ± 40.45	386.15 ± 96.1	184.25 ± 70.22	240.65 ± 189.86	210.05 ± 28.5	208.6 ± 113.14
A_260/280_	1.08 ± 0.04	1.37 ± 0.04	1.51 ± 0.08	1.38 ± 0.05	1.15 ± 0.04	1.31 ± 0.08	1.46 ± 0.02	1.18 ± 0.13	1.1 ± 0.01	1.47 ± 0.03
A_260/230_	0.25 ± 0.03	0.46 ± 0.04	0.79 ± 0.13	0.48 ± 0.06	0.26 ± 0.07	0.27 ± 0.02	0.57 ± 0.00	0.25 ± 0.08	0.3 ± 0.02	0.39 ± 0.01
6 Salting out method (S)										
DNA yield (ng/µl)	62.2 ± 26.87	1701.8 ± 1046.80	3599.4 ± 1363.87	327.5 ± 286.38	118.45 ± 23.83	581 ± 50.91	3264.4 ± 3135.45	1169.75 ± 582.16	246.85 ± 24.96	1248.4 ± 792.67
A_260/280_	1.95 ± 0.04	1.81 ± 0.07	1.81 ± 0.04	1.97 ± 0.13	1.95 ± 0.16	1.78 ± 0.04	1.84 ± 0.06	1.83 ± 0.4	1.83 ± 0.04	1.83 ± 0.11
A_260/230_	0.9 ± 0.51	0.61 ± 0.3	0.96 ± 0.01	0.37 ± 0.41	0.15 ± 0.04	0.68 ± 0.03	0.77 ± 0.40	0.48 ± 0.25	0.58 ± 0.11	0.46 ± 0.29

### Evaluation of DNA integrity by agarose gel electrophoresis

**Fig. 1:**
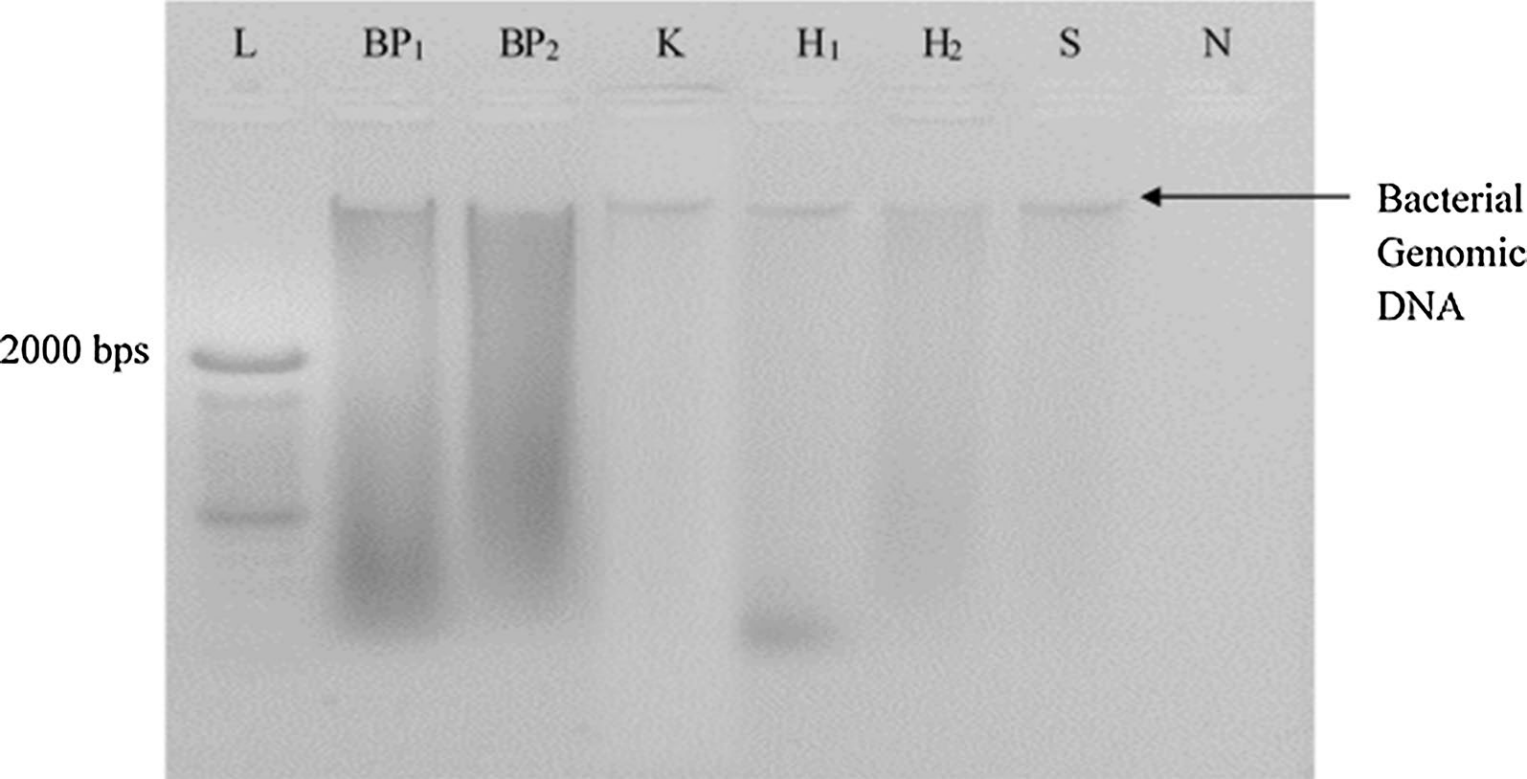
The quality of DNA extracted from wound tissue debridement specimen No. 1 using six DNA extraction methods

The integrity of extracted DNA samples was analyzed by agarose gel electrophoresis. A high molecular weight band indicated the presence of genomic DNA (Fig. 1). All six DNA extraction methods showed low intense bands on agarose gel (Fig. 1). However variation in the yield and purity of DNA were observed among different methods.

When the bead beater–phenol chloroform extraction methods were occupied (BP1 and BP2 method) BP1 method yielded maximum DNA yield and high A260/280 nm ratios (1.8–2.0) in each specimen. Of the ten specimens, only four yielded A260/280 nm ratios >1.8. Comparatively to BP1 method, BP2 method yielded low DNA amount with a little contamination by the respective substances.

The two heating methods including H1 and H2 yielded considerable amount of DNA with more contamination of proteins and other impurities. DNA extraction using S method could yield maximum amount of DNA from most of the specimens but, their purity was very low. The reason for the lower DNA purity may be due to possible high salt contamination. DNA extraction using K method yielded considerably high DNA yield with a maximum A260/280 and A260/230 ratios indicating minimal DNA contaminants.

Following the Welch statistics, the extraction methods which have significant differences between the mean values of their DNA yield were grouped and multiple comparison was done using the Games–Howell. According to the multiple comparison, it was found that there was not a significant difference (at 95% confident level) between the means of DNA yield produced by each method. However, it was found that there was a significant difference (at 95% confident level) between the means of A260/280 ratio (p = 0.000): BP1 method and H1, H2 methods, BP2 and H1, H2 methods; H2 and K methods; S and H1, H2 methods. Further, a significant difference (at 95% confident level) was found between the means of A260/230 ratios (p = 0.000): BP1 method and H1, H2, S methods; BP2 method and H1, H2, S, K methods; K method and H1, H2, S methods.

### PCR amplification of V2–V3 region of 16S rDNA

**Fig. 2:**
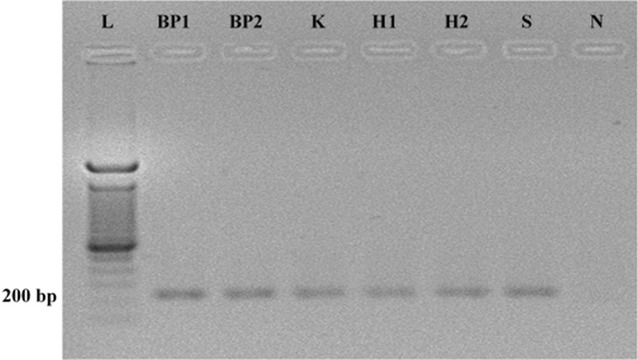
Agarose gel electrophoresis of PCR amplified product of V2–V3 region of 16S rRNA gene from bacterial genomic DNA which was extracted from wound tissue debridement specimen No. 1 using different extraction methods. *L* 100 bp DNA ladder marker, *N* blank/Negative control

In order to evaluate the effect of DNA extraction method on the quality of DNA, the DNA extracted from all six methods were used for the determination of the microbial biodiversity in wound specimens. PCR amplification of the V2–V3 region of extracted bacterial 16S rRNA gene was carried out and the products were run on agarose gel (Fig. 2). The agarose gel electrophoresis of PCR product showed well separated and consistent bands with no impurity bands, with a product size of approximately 200 bp indicating the presence of well amplified V2–V3 region of 16S rRNA gene of bacterial species (Anukam and Reid 2007; Oates et al. 2012; Walter et al. 2001).

### Comparative analysis of DGGE fingerprint profiles

**Fig. 3:**
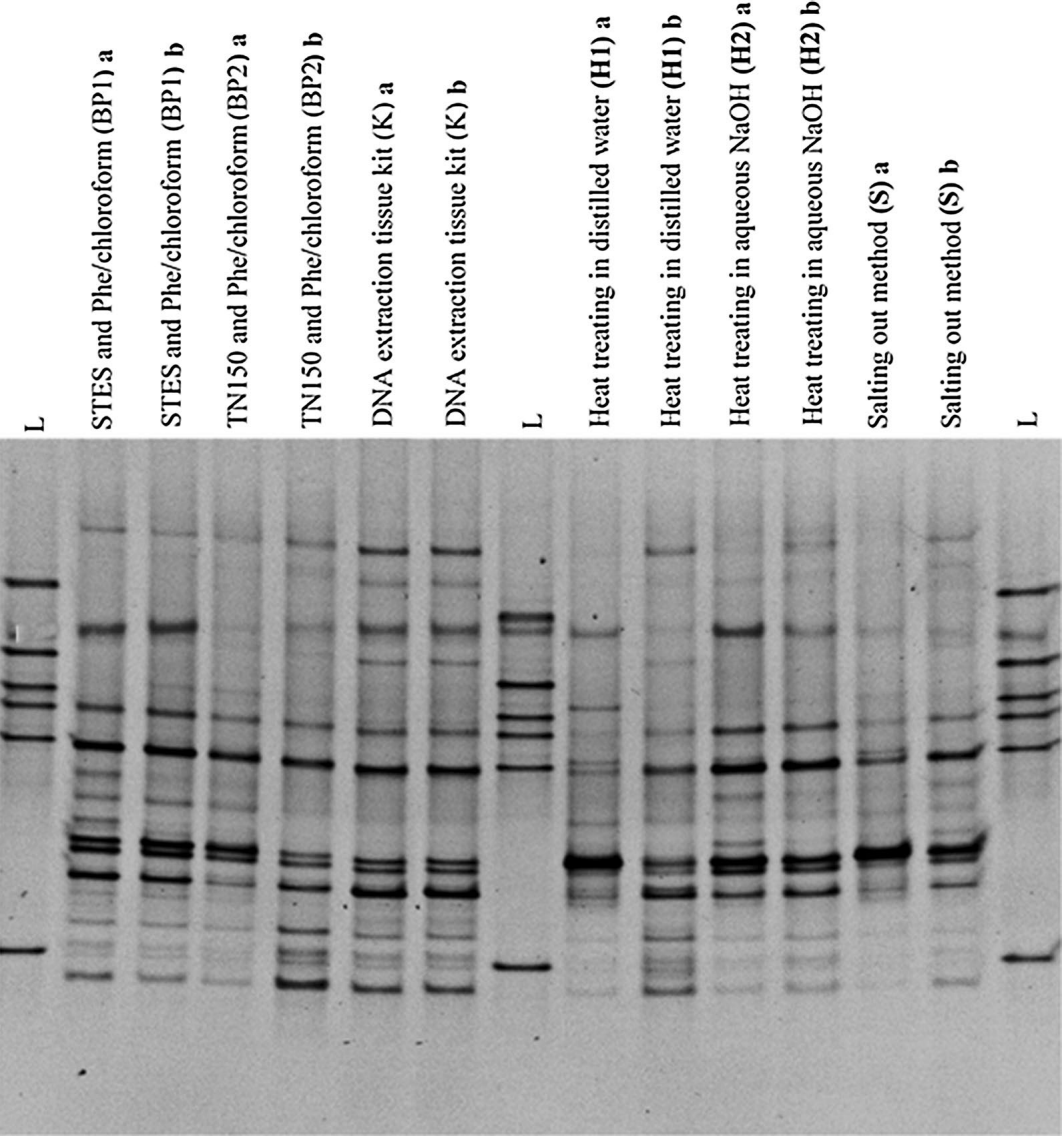
A comparison of DGGE profiles of PCR amplified bacterial 16S rRNA gene for the specimen No: 1. DNA was extracted using six different DNA extraction methods using 25 mg of wound tissue debridement specimen no. 1. Bacterial fingerprinting profile is based on 30–55% denaturing gradient. “L” lanes represent the in house bacterial reference panel which includes *S. aureus, Acinetobacter* spp, Group B *Streptococcus* spp., *E. faecalis*, Group A *Streptococcus* spp. and *E. coli* from top to bottom respectively. Other lanes show bacterial fingerprinting profile of each extraction method in duplicate (a, b) for the specimen No. 1, collected from a subject with a chronic wound

**Fig. 4:**
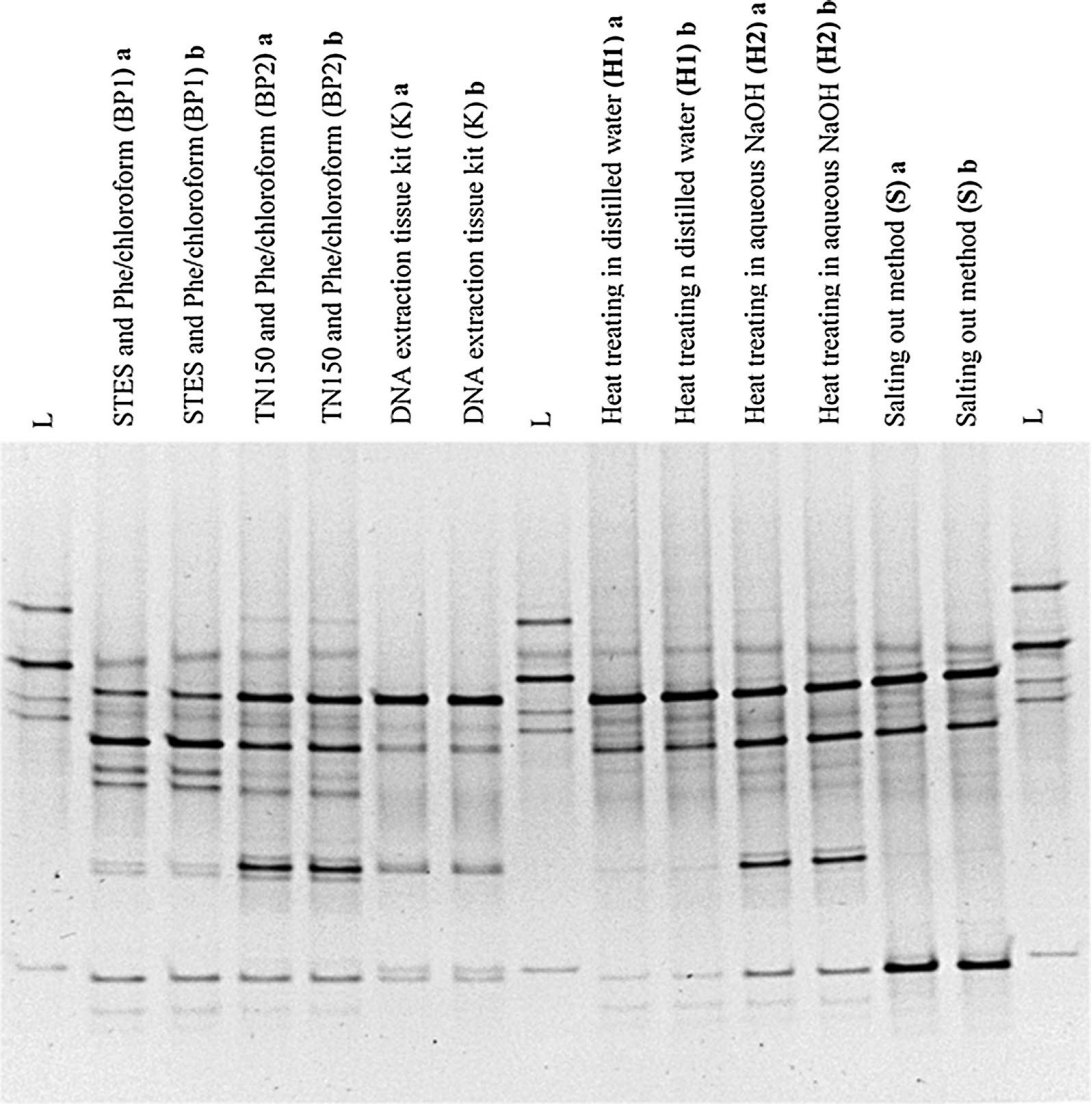
A comparison of DGGE profiles of PCR amplified bacterial 16S rRNA gene for the specimen No: 2. DNA was extracted using six different DNA extraction methods. Bacterial fingerprinting profile was based on 30–55% denaturing gradient. “L” lanes represent the in house bacterial reference panel which includes *S. aureus, Acinetobacter* spp., Group B *Streptococcus* spp., *E. faecalis,* and *E. coli* from top to bottom respectively. Other lanes show bacterial fingerprinting profile of each extraction method in duplicate (a, b) for the specimen No. 2, collected from a subject with a chronic wound

Highly diverse DGGE banding patterns were observed in each specimen on 30–55% denaturing gradient. Further intra- and inter-subject variations were readily observed in the specimens following different extraction methods (Figs. 3, 4). The differences in DGGE profiles were reflected by the number and intensity of the DNA bands in comparisons of bacterial profiles of the same sample using different DNA extraction methods (Figs. 3, 4). Each DGGE band was assumed to represent a single species (Muyzer et al. 1999). Therefore, the number of bands from each lane was counted and average number of bands per specimen was calculated (Table 3).

**Table 3:** Determination of microbial diversity of six different extraction methods using DGGE

DNA extraction method	Average number of bands in each specimen									
	S/no: 1	2	3	4	5	6	7	8	9	10
STES buffer and phenol chloroform extraction method (BP1)	14	10	06	08	16	11	06	04	04	08
TN150 buffer and phenol chloroform extraction method (BP2)	14	12	07	09	13	12	07	05	08	09
DNA extraction tissue kit (K)	15	06	07	10	11	13	07	04	05	06
Boiling in distilled water (H1)	13	09	05	08	06	08	05	05	08	05
Boiling in aqueous NaOH (H2)	14	11	06	11	10	12	07	06	08	07
Salting out method (S)	14	09	05	07	10	12	05	05	08	06

*S/No* specimen number

The observed multiple bands in the DGGE gel are suggestive of a polymicrobial nature of the chronic wound infection. Theoretically, all profiles should have been identical since DNA was extracted from the tissues obtained from same chronic wound specimen. However, there was a variation in intensity of bands and number of the bands in the same specimens which were subjected for different DNA extraction methods. This observation suggests that the different DNA extraction methods may have had varying sensitivity for the different bacteria.

### Excising of selected DGGE bands, PCR amplification, and sequencing

Selected predominant DGGE bands from the DGGE gel were excised, PCR amplified, purified and sequenced using Sanger method. According to the sequencing results, *Enterococcus feacalis*, Group A *Streptococcus* spp., *Providencia* spp., *E.coli* and *Vellionella* spp. were found in the sample no. 1 following six extraction methods (Fig. 1). However the resulting intensity of these DGGE bands differed in different extraction methods. *Pseudomonas aeruginosa* was found from six extraction methods done for specimen No. 2 (Fig. 2). When considering the specimen No. 2, *Providencia* spp. could only be identified from the BP1 method while other methods did not yield the specific DNA band (Fig. 2).

## Discussion

Among the five tested conventional methods, bead beater/phenol–chloroform based DNA extraction method with STES buffer (BP1 method) gave a yield of DNA with high purity. Further it resulted in broad diversity of bacterial communities in chronic wounds using PCR-DGGE. This method is a less time consuming process and less expensive, therefore it can be easily applied for the settings where expediency and cost effectiveness is essential. This reinforces the BP1 method as an appropriate, conventional DNA extraction method to study microbial communities in human chronic wounds. The DNeasy Blood and Tissue Kit (K method) produced good quality genomic DNA with appreciably greater yield compared to the quality and yield of the other five conventional DNA extraction methods. Further K method gave a broad diversity of bacterial communities in chronic wounds using PCR-DGGE.

Isolation of genomic DNA is a multi-step procedure including cell disruption, DNA extraction and DNA recovery. Bacterial cell lysis is a critical step in the extraction process. The extracted DNA should be free of contaminants including the least amount of proteins, carbohydrate, lipids, other nucleic acid (RNA), other cellular constituents that may interfere with restriction enzymes, ligase and thermostable DNA polymerases or any other PCR inhibitors (Asadzaheh et al. 2010). Removing contaminants is one important key factor for a successful PCR, since quality and integrity of the isolated DNA will directly affect the results of all succeeding procedures.

Absorbance of DNA was measured at 260 nm to evaluate the quantity of the extracted DNA, and the ratio of A260/280 nm was used to evaluate the DNA purity. The A260/230 nm ratio is used as a secondary measure of nucleic acid purity. The A260/230 values for “pure” nucleic acid are often higher than the respective A260/280 values. Commonly expected A260/230 ratio should be greater than 1.8. If the ratio is appreciably lower than expected, it may indicate the presence of contaminants which absorb at 230 nm such as TE buffer.

Small changes in the pH of the DNA solution can cause variations in A260/280 ratio (Wilfinger et al. 1997). The A260/280 ratio will be under-represented and over-represented by 0.2–0.3, when DNA are in acidic solutions and basic solutions respectively.

Commonly used DNA extraction procedures employ a buffer containing one or several detergents such as SDS, NP-40, Triton X–100 or CTAB which aids cell lysis and the removal of proteins from DNA (Moore and Dowhan 1995). Both STES and TNES buffers used for the DNA extraction in our study (BP1 and S methods) contains SDS. Excessive SDS above 0.01% may inhibit the PCR by denaturing Taq polymerase (Yang 2008). Therefore, extracted DNA had to be diluted 1:10 with PCR water before the PCR amplification. In our study, the DNA yield obtained from BP1 method is ranged 49–1747.95 ng/µl and A260/280 ratios for all specimens were greater than 1.8. However A260/230 ratios for several specimens resulted less than 1.8 indicating residual carryovers such as phenol, in the extraction mixture. The DNA yield obtained by S method (ranged 62–3599 ng/µl) was greater than the DNA yield obtained by other five methods. Further, A260/280 nm ratios for all specimens were similar or greater than 1.8 but, A260/230 nm ratios were always less than 1.8 indicating a high salt contamination in the extraction mixture. In the S method, the use of saturated NaCl results in protein precipitation followed by DNA precipitation by ethanol (Yang 2008). Although washing with 70% ethanol would remove the residual carryovers, presence of high salt contamination in the extraction mixture can result in a decrease in purity of DNA.

When considering phenol–chloroform based extraction methods (BP1 and BP2 methods) the DNA quantification and purity determination using UV absorbance at 260 nm may be error prone due to the high extinction coefficient of phenol at 260 nm resulting in lower detectable yields (Yang 2008). Further, the toxicity of phenol and labor-intensity should be carefully considered when phenol–chloroform based extraction methods are carried out. Therefore, this method can be further improved by using a phase lock gel as a barrier.

In the current study, BP1 method yielded good quality DNA compared to BP2 method. A lower A260/230 nm ratio may be due to phenol contamination in the extracted mixture. Under BP2 method, several specimens out of ten specimens resulted the A260/A280 nm ratio as less than 1.8 that indicates protein contamination or presence of organic contaminants, such as phenol, and other aromatic compounds.

In this study, two different heat treatment methods were followed using sterile distilled water and aqueous NaOH. DNA is less reactive and stable in alkaline conditions. Therefore it is very important to avoid acidic conditions during DNA extraction, since depurination of DNA can occur. Therefore, DNA is usually stored at pH 8.0 to avoid even slightly acidic conditions that may over time lead to base losses. In this study, when the extractions were done using the heating methods such as H1 and H2, both A260/A280 nm ratios and A260/230 nm ratios for all ten specimens were less than 1.8 that was indicative of ominously low purity/quality DNA due to the absence of specific DNA purification steps following cell lysis. Therefore although these two methods were extremely easy and inexpensive, the DNA purity was very poor.

In DNeasy blood and tissue kit method, de-proteinization was achieved by proteinase K. Proteinase K is active in the presence of SDS and also at elevated temperature (56–65 °C). However other enzymes such as DNases are denatured under these conditions. In our study, K method yielded good quality DNA than other five methods.

As many reports described, commercially available mini column-purification methods yield DNA of high purity containing the least amount of PCR-inhibitory substances. Oates et al. (2012), reported extraction of DNA from archived macerated chronic wound tissue samples and swab samples using DNeasy blood and tissue kit for microbial profiling using PCR-DGGE (Oates et al. 2012). This method enabled DNA extraction in less than 3 h.

DNeasy blood and tissue kit resulted in the largest diversity of bands in PCR-DGGE followed by the BP1 method. Although the H2 method had the lowest purity DNA, a good band diversity was observed in DGGE gel.

In this study, the identification of the different bacterial species in each specimen was not done using PCR-DGGE. The objective was to evaluate effective DNA extraction method to profile the bacterial population in the infected chronic wounds using PCR-DGGE. The DGGE method is a qualitative and semi-quantitative method; the abundance of microorganisms can be reflected by the DGGE profile (Fan et al. 2014). The number of DGGE bands can reflect the bacterial diversity of the infected chronic wounds, and can be applied for semi-quantitative analysis using the intensity of the bands.

Each extraction method had their own pros and cons. Many variations can be found in DNA extraction methods which occur between laboratories. This may diminishes the consistency and comparability between studies. These findings suggest that the quality and yield of genomic DNA is influenced by each DNA extraction protocol.

## Abbreviations

DNA: deoxyribonucleic acid
PCR: polymerase chain reaction
DGGE: denaturing gradient gel electrophoresis
RNA: ribonucleic acid
NaOH: sodium hydroxide
rDNA: ribosomal deoxyribonucleic acid
HCl: hydrochloric acid
EDTA: ethylenediaminetetraacetic acid
SDS: sodium dodecyl sulfate
TE: Tris-EDTA buffer solution
MgCl2: magnesium chloride
dNTP: deoxy nucleoside tri-phosphate
dATP: deoxy adenosine tri-phosphate
dGTP: deoxy guanidine tri-phosphate
dCTP: deoxy cytosine tri-phosphate
dTTP: deoxy thymidin tri-phosphate
TAE: Tris base, acetic acid and EDTA
UV: ultra violet
SPSS: statistical package of social sciences
SD: standard deviation
CTAB: cetyl trimethylammonium bromide
NaCl: sodium chloride
