# Correction to: Evaluation of the impact of six different DNA extraction methods for the representation of the microbial community associated with human chronic wound infections using a gel‑based DNA profiling method

**DOI:** 10.1186/s13568-020-01145-w

**Published:** 2020-11-26

**Authors:** Ayomi Dilhari, Asanga Sampath, Chinthika Gunasekara, Neluka Fernando, Deepaka Weerasekara, Chris Sissons, Andrew McBain, Manjula Weerasekera

**Affiliations:** 1 Department of Microbiology, Faculty of Medical Sciences, University of Sri Jayewardenepura, Gangodawila, Nugegoda Sri Lanka; 2 Department of Surgery, Faculty of Medical Sciences, University of Sri Jayewardenepura, Gangodawila, Nugegoda Sri Lanka; 3 Department of Pathology and Molecular Medicine, University Otago, Wellington, New Zealand; 4 Faculty of Biology, Medicine and Health, The University of Manchester, Manchester, M13 9PT UK

## Correction to: AMB Expr (2017) 7:179 https://doi.org/10.1186/s13568-017–0477-z

Following publication of the original article (Dilhari et al. 2017), the authors identified an error in Figs. 1 and 3.

The corrected figures are given below.

**Fig. 1:**
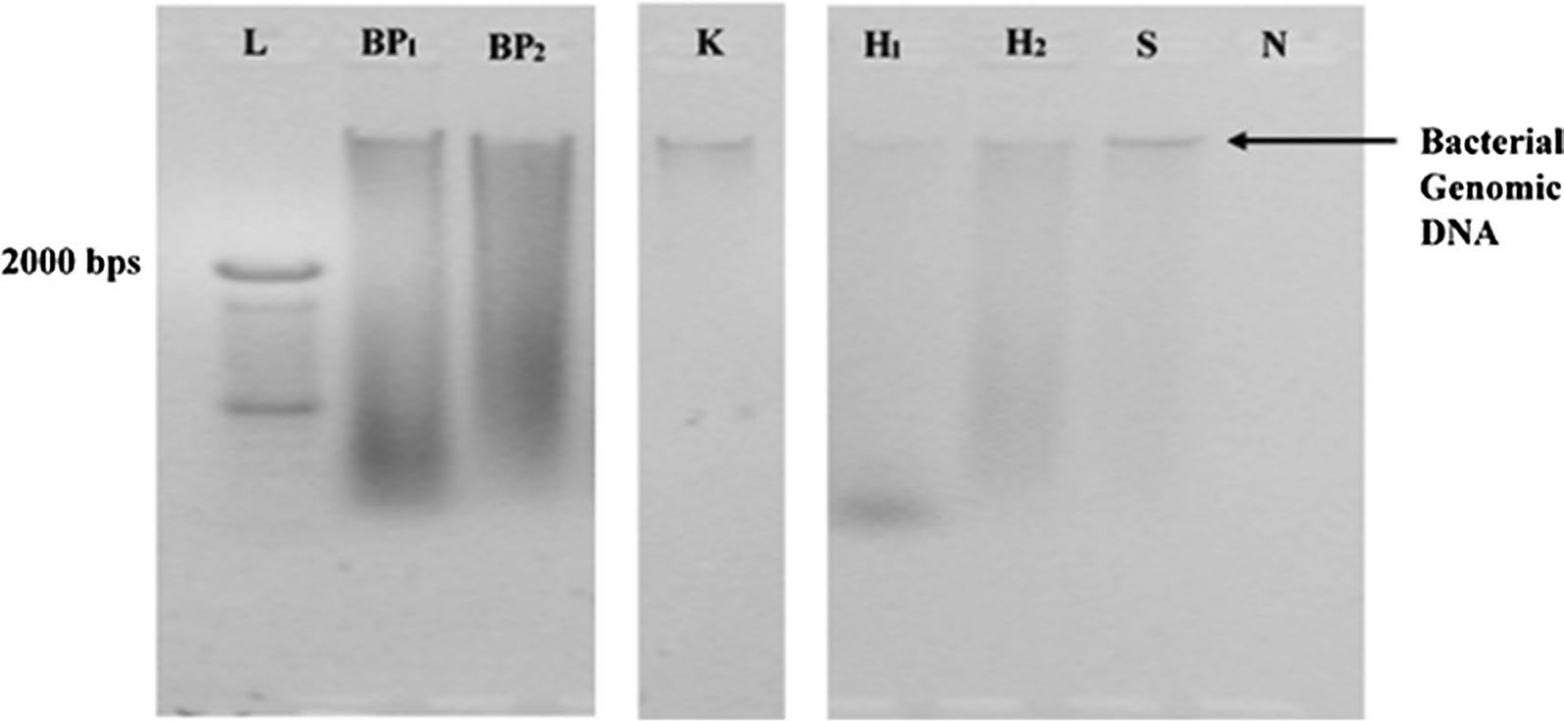
The quality of DNA extracted from wound tissue debridement specimen No. 1 using six DNA extraction methods

**Fig. 3:**
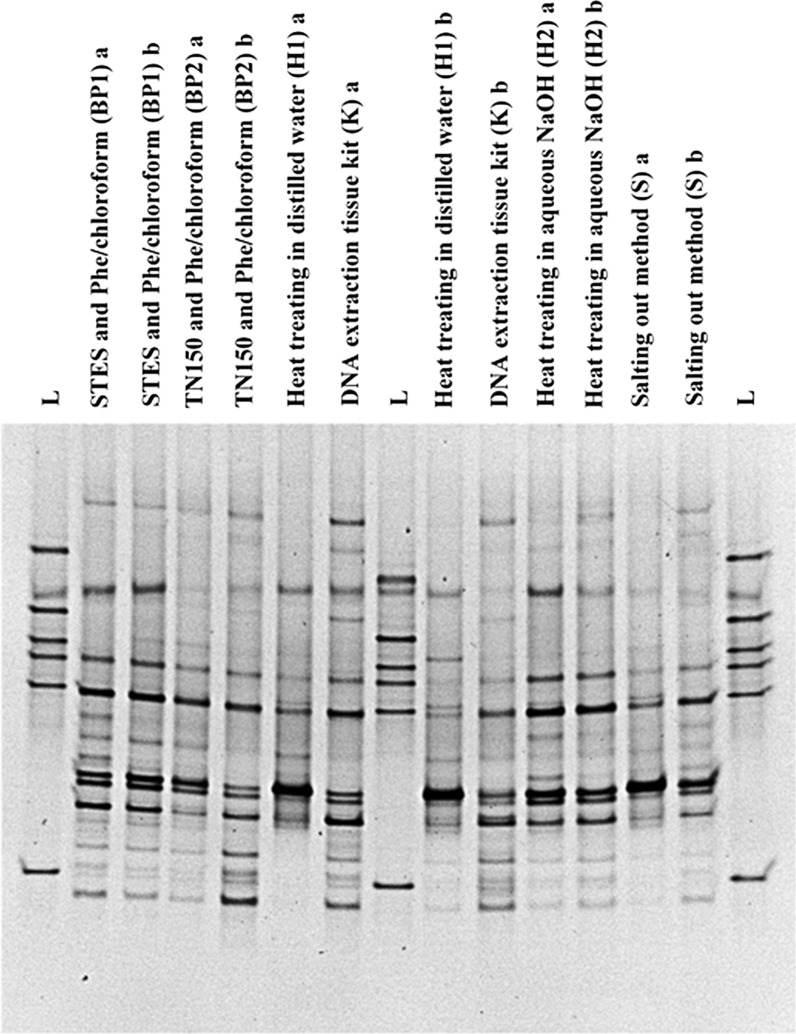
A comparison of DGGE profiles of PCR amplified bacterial 16S rRNA gene for the specimen No: 1. DNA was extracted using six different DNA extraction methods using 25 mg of wound tissue debridement specimen no. 1. Bacterial fingerprinting profile is based on 30–55% denaturing gradient. “L” lanes represent the in house bacterial reference panel which includes *S. aureus, Acinetobacter* spp, Group B *Streptococcus* spp., *E. faecalis*, Group A *Streptococcus* spp. and *E. coli* from top to bottom respectively. Other lanes show bacterial fingerprinting profile of each extraction method in duplicate (**a**, **b**) for the specimen No. 1, collected from a subject with a chronic wound
